# Some Characteristics

**Published:** 1887-09

**Authors:** 


### SOME CHARACTERISTICS.

IODIUM.—Purulent stools, with cutting pains in the intestines, nausea and vomiting, and sour taste in the mouth. Have cured several such cases with single doses of the highest potencies.

Vertigo, throbbing in the head and all over the body, tremor at the heart and fainting, worse after just rising from a seat or bed, or on sitting or lying down after slight exercise; very sickly look of the patient.

Subsultus tendinum of both hands and feet, great drowsiness and continual dreaming of eating, with great prostration on rising from bed, and on lying down again, picking of bedclothes and short, dry cough.—*M. Preston.*

RHODODENDRON AND RHUS.—Both remedies have rheumatic pains, especially in all the aponeuroses; worse when at rest; worse at night.

1. *Rhodod.*—Pains do not admit of the limbs being at rest; desire to move, and moving relieves. (F. Husmann, C. Hg.)

2. *Rhus.*—Rest occasions uneasiness in the painful parts, but, on moving, the pain is worse. (C. Hg., Neidhard.) Continued motion only relieves.

3. It is known that *Rhod.* has general aggravation of pains before a change in the weather—particularly before a thunder storm—even in dysentery indicated by this. (C. Hg.)

4. *Rhus.*—Has aggravation from the warmth of the bed, and as a general characteristic in consequence of stretching, over-lifting, over-exertion of joints, etc., or from getting wet while perspiring.

5. *Rhod.*—Acts more on the right side, and, according to Bœnninghausen, *Rhus* more to the left.

6. There is not much known about the direction of either; or which side is first affected, or which afterward. Cases cured would be worth recording, if the order of sides had been observed. Provers ought to do the same.

7. *Rhod.* has aggravation of pains in the night, but more toward morning; *Rhus*, more toward evening and night.

8. *Rhus* corresponds to rheumatism in the cold season; *Rhod.* in the hot season. *Rhod.* worse before, and *Rhus* worse after rain.—*Homœopathic News.*

Stramonium.—Vertigo, when walking in the *dark*, day or night. When walking in the dark at night, he staggers and falls down every time he attempts to walk. The same occurrence transpires when he attempts to walk in a darkened room in the daytime.

Several years since, I cured with *Stram.* a case of this kind, of several months' standing, after all sorts of treatment in the old school had utterly failed. He was a wealthy man, a good liver, and very corpulent or plethoric. I was induced to administer as I did from the fact, "Moral symptoms aggravated in the dark." Why not the physical also?

Young *men* are cured, as well as young women, when they pray, sing, or talk in a very devout, earnest, and constant manner, so as to excite the sympathy of all in the house. This has been my experience.

In typhus, typhoid, or other fevers, when the patient frequently raises or jerks the head from the pillow. An old key-note, and sure; one of the most characteristic.

All sorts of strange and absurd ideas, such as the patient is double and is lying crosswise.

The only new idea in this paper or note is the fact of falling in the dark always, but can walk well in the light. I have had occasion to observe this condition but once. I presume, though, it will prove a reliable symptom. The mental aggravation in the dark I am familiar with; perhaps it remains to be confirmed in regard to the physical. He seemed unable to tell me why he fell, but if he wanted to go out in the night, or after dark, a strong man had to walk on each side of him to keep him up.—*H. N. Guernsey.*

STANNUM.—Everything in the street, in advance of the patient, seems to be up in the air. The whole street appears to be in the air. The patient takes high steps in walking in order to get up to the level of the apparent height of the objects (as if the chair were rising; Phosp.).—*Guernsey.*

